# On Etherization in Labor

**Published:** 1848-03

**Authors:** 


*On Etherization in Labor.* [Read before the Boston Society for Medical Improvement, Dec. 27th, 1847, by C. G. Putnam, M. D.] The cases which form the subject of remark are nineteen in number. I have included no case in which the patient was not, for a longer or shorter period, decidedly under the influence of ether. I watched them with more than ordinary attention, and, as I have reason to believe that the experience of other gentlemen in this place corresponds with my own, the results will aid us in making an estimate of its merits. I have selected a few cases which exemplify the points of most interest.

The first is that of a lady 23 years of age. First gestation. Health robust. Presentation vertex. Length of labor twenty-four hours. The dilatation of the os uteri was attended with unusual distress and nervous agitation. I proposed the use of ether, and she gave it a single imperfect trial. The result was unsatisfactory. It caused nausea and sensations of faintness and confusion in the head, and she preferred to go on without it. The expulsive pains were violent, and towards the close of the labor I again offered it. She happened to be breathing deeply when the sponge was applied, and three or four inspirations were sufficient to give entire relief. The transition from a state of restlessness and agitation to that of ease and repose, was immediate. She retained the sponge for a few minutes longer, when the child was born and she was laid comfortably in bed—the whole process having been accomplished, not indeed without her knowledge, but without scarcely a sensation of inconvenience.

Case II.—Presentation of nates. A lady, æt. 24, rather below medium size, constitution delicate. First gestation. During the last two months of gestation had anasarca of the lower extremities, accompanied with sensations of great distension of the abdomen. Restless and uncomfortable day and night. She became much reduced in health and spirits. After two or three days of precursory pain, the dilatation of the os uteri commenced. This stage was exceedingly tedious and irksome. After free dilatation, the presenting part remained at the brim of the pelvis, and thirty-six hours elapsed before it had descended into the cavity. The membranes, up to this time, had fortunately remained unbroken.

The pains, which from the first had been slight, short and insufficient, now made scarcely any impression. It was evident that artificial aid was necessary, and as soon as practicable I succeeded in passing a cord round the thigh. To this a soft well-larded handkerchief was attached and drawn up round the groin. Fifteen grains of ergot were given, gentle traction made during every pain, and in about an hour the child was delivered living. Its weight was nine pounds. The uterus contracted at once, and expelled the placenta, but in a few minutes it relaxed and considerable internal hemorrhage ensued. On removing the coagula, contraction again took place, and there was no further relaxation.

Ether was used sparingly in this case, on account of the deficiency of the pains. Whenever applied, its effects were manifested after inhaling for about a minute, and almost invariably soothed irritability and promoted repose. At the close of the labor it was carried to the extent of partial insensibility.

Notwithstanding the nature of the presentation and the length of the labor, the secretions were abundantly maintained, and there was at no time either heat or dryness. Convalescence was retarded by mammary abscess. The child was healthy.

Case III.—A lady, 22 years of age. Healthy and strong. First gestation. Presentation vertex. Length of labor, eighteen hours. I was called early, and found the os uteri soft and dilated about half an inch. She had been in pain four hours, and having become uneasy she eagerly made trial of ether. In less than two minutes she became quiet, but not unconscious. At each access of pain she asked for the sponge, and retained it till she was easy. Administered in this manner at the beginning of labor, it was continued at short intervals throughout the first stage, and contributed much to the relief of the wearisome pains of dilatation.

At the commencement of the expulsive stage, I observed that the efforts were suspended, and the process so much retarded by the inhalation, that it was often necessary to withhold it, much to the dissatisfaction of the patient. When, however, the head began to press upon the perineum, the sponge was applied without reserve, and she was unconscious for the half hour preceding and following the birth of the child. The contraction of the uterus was immediate and thorough. The placenta removed at once. The convalescence uninterrupted. The child very healthy.

Case IV.—A lady, æt. 23. First gestation. Health good. Head presentation. Length of labor, twelve hours. I was not called until the beginning of the expulsive stage. The pains occurred every four or five minutes. They were strong, but not remarkably severe.

After inhaling for two minutes, she passed into a state of pleasant excitement and exhilaration. She was conscious of pain, but "it was so far off" that she did not regard it. "It was no concern of hers." Conscious of everything that was passing around her, and of questions addressed her, she felt at the same time incapable of responding, though she talked with great volubility of herself and her condition. One would have inferred from the apparent consciousness during the pains and the accustomed straining efforts, that she was suffering as much as usual—but on the contrary the close of each "pain" was announced by an extravagant eulogium upon ether and its inventors.

She continued to inhale in this manner for two hours without obvious diminution of the force or frequency of the parturient efforts. Just at the close the sponge was applied assiduously, and, though at no time unconscious, she did not realize the least severe pain. The contraction of the uterus was thorough and immediate. The placenta removed at once. Convalescence perfect.

Case V.—I have applied the forceps, under full etherization, in three cases. The conditions in all were essentially the same. I will therefore relate only the last, which occurred the present week.

I was called to this case by a medical friend, who informed me that she had been in active labor for thirty-six hours, that the head had descended into the cavity of the pelvis, where it had remained for some hours immovable, though the pains had been constant and strong. Age 30. First gestation.

During the whole labor she had been clamorous for ether, and it had been administered incessantly, though not to the full extent.

During the delivery—a space, perhaps, of half an hour—she was kept almost entirely unconscious. The child weighing nine pounds, was born living. The uterus contracted well, and the placenta was thrown off at once.

The breath of the child smelt very strongly of ether—more so than in any case that I have met with.

In all of these cases the mother and children did well.

Case VI.—*Arm presentation.* "Waters" discharged and arm protruded *four* days. Pains constant.

A professional friend, who asked me to assist him, stated that he had just been called to the case, and found her in the condition above described. He had made an attempt to "turn" but had relinquished it. When I saw her, the pains were strong. The protruded arm livid, but the fœtal pulsations were distinct.

After inhaling for two or three minutes, she became furiously excited, and was restrained with considerable difficulty. Repose being essential to the success of the operation, we persisted in the application of the sponge well filled with ether for about six minutes, when she relapsed into a state of utter unconsciousness. I was then able to pass my hand through the os uteri, and reached the feet with very little effort, and without being in the least degree cramped. There was no liquor amnii in the cavity, and the uterus was closely applied to the unequal surfaces of the fœtus. A foot was brought down to the os uteri, and a tape looped round the ancle. By drawing upon this with the right hand, and at the same time rotating the femur with the left, the arm began to recede, and the evolution once begun, was readily completed. The child was delivered living; the placenta thrown off, and removed at once. During the whole time of delivery—little more than half an hour—she was motionless and unconscious, and yet the child was scarcely washed when she insisted on sitting up in bed to give directions about its dress.

Etherization was here carried to its full extent, and the effects were in the highest degree important and gratifying.—*Boston Med. and Surg. Journal.*

